# Visually-induced dizziness is associated with sensitivity and avoidance across all senses

**DOI:** 10.1007/s00415-020-09817-0

**Published:** 2020-04-18

**Authors:** Georgina Powell, Hannah Derry-Sumner, Katherine Shelton, Simon Rushton, Craig Hedge, Deepak Rajenderkumar, Petroc Sumner

**Affiliations:** 1 School of Psychology, Cardiff University, Tower building, Park Place, Cardiff, UK; 2 Department of Audiovestibular Medicine, University Hospital of Wales, Cardiff, UK

**Keywords:** Persistent postural perceptual dizziness (PPPD), Visually-induced dizziness, Visual stress, Sensory overload, Visual vertigo, Migraine

## Abstract

**Background:**

Persistent postural perceptual dizziness (PPPD) is a common chronic condition presenting in neurology and neuro-otology clinics. Symptoms lie on a spectrum in the general population. The cause is unknown and thought to involve interactions between visual and vestibular systems, but symptoms also correlate with anxiety and migraine.

**Objective:**

To test whether PDDD symptoms are associated with reported differences in other senses (touch, hearing, smell and taste); to investigate possible mediation via anxiety or migraine; to discover the proportion of variance accountable to these non-vestibular factors.

**Methods:**

We measured self-report multisensory sensitivity, anxiety, visual difficulties, visual discomfort and migraine in patients with PPPD (*N* = 29) and a large general population cohort (*N* > 1100). We used structural equation modelling to examine relationships between the factors using a step-wise approach.

**Results:**

We found increased self-reported over-sensitivity in sensory domains beyond vision and balance in both patients with PPPD and non-clinical participants with more PPPD symptoms. SEM analysis revealed that anxiety partly, but not wholly, mediated this relationship. Adding visual difficulties and visual discomfort to the model allowed it to explain 50% of PPPD symptom variance. Most of the path coefficients and mediation effects in our model were unchanged between participants with and without migraine.

**Conclusions:**

Our findings support the idea that PPPD is a complex neurological condition that includes broad perceptual factors, and may suggest that some brains are predisposed to generalised cross-modal sensory-overload. This may give rise to vulnerability to severe PPPD should a vestibular insult occur.

**Electronic supplementary material:**

The online version of this article (10.1007/s00415-020-09817-0) contains supplementary material, which is available to authorized users.

## Introduction

Persistent postural perceptual dizziness (PPPD) is a chronic and debilitating condition characterised by dizziness and vertigo in response to upright posture, self-movement and visual motion [1–3]. It is the second most common diagnosis in patients reporting dizziness [4] and is a common complaint in neurology and neuro-otology clinics. Challenging visual environments such as supermarkets, cinemas and busy roads are frequent triggers for PPPD symptoms [1, 5]. Patients with PPPD often first report with a vestibular insult such as labyrinthitis, but their symptoms remain even once the peripheral disorder resolves [1]. This suggests that the root dysfunction in PPPD is central rather than peripheral.

Prevailing theories of PPPD suggest that the strategies (conscious or unconscious) that patients employ to compensate for their peripheral disorder persist after recovery and become maladaptive. These include a tendency to rely on visual information over vestibular for postural stability [termed ‘visual dependence’; 6, 7–10] and a tendency to be over-vigilant about the vestibular sensations that accompany self-actions [11, 12]. Patients can also develop functional gait abnormalities, likely in an effort to reduce symptoms [2].

PPPD is strongly associated with psychogenic factors such as anxiety and panic disorder, often to a greater extent than other vestibular conditions [13–16]. Anxiety can be a predisposing factor in developing dizziness [17, 18], and some neuro-otology patients go on to develop a secondary anxiety disorder during their vestibular disease [19–21]. There is likely to be a reciprocal and self-perpetuating link between the two, where dizziness and postural instability lead to anxiety due to fear of falling and nausea, and high anxiety states induce more dizziness symptoms [13, 15, 21–25]. Anxiety is associated with worse outcomes for patients with PPPD [e.g. 26] and clinicians often emphasise the importance of treating anxiety symptoms alongside, or even before, dizziness symptoms.

We have recently reported that sub-clinical symptoms of PPPD are common in the general population, with around 10% of individuals scoring above the patient 25th percentile on common questionnaire measures of PPPD [27]. As with patients reporting to clinics, the symptoms were sometimes associated with migraine and anxiety, but these associations did not fully explain the spectrum. We proposed that some individuals are predisposed to experiencing visually-induced dizziness in everyday life even without any historical vestibular deficits. This predisposition would then make full PPPD more likely to develop if they suffer a vestibular insult.

### PPPD and multi-sensory processing

Although PPPD is normally associated with moving stimuli or self-movement, we have found that patients with PPPD, and individuals in the general population with more symptoms of PPPD, report increased visual discomfort/stress when viewing static images that deviate from the statistical properties of natural scenes [28]. Increased visual discomfort is also found in migraine and has been associated with over-activity in the visual cortex and reduced visual discrimination thresholds [29–33]. Importantly, however, migraine did not explain the association with PPPD symptoms [28]. Low vision ability is also associated with a general increase in dizziness [see 34, for a review]. These findings emphasise the importance of considering the visual component of PPPD, alongside vestibular and psychogenic factors.

Furthermore, our anecdotal experience of patients with PPPD is that they often report feelings of ‘sensory overload’ – an inability to attend to and focus on the world around them, and a general oversensitivity to sensory information. This led us to hypothesise that PPPD could be associated with multi-modal sensory abnormalities that go beyond vision, vestibular and proprioception. Such global sensory differences have been reported in other conditions such as autism spectrum disorder [ASD; 35, 36], obsessive-compulsive disorder [OCD; 37] and Schizophrenia [38]. There is also evidence that patients with PPPD show differences in pain habituation and thresholds, which could be evidence of a more widespread central perception dysfunction [39].

A common framework for assessing multi-sensory differences in clinical disorders is Dunn’s [40] model of sensory processing. According to this model, individual differences in sensory experience fall along two continuums, which may map on to individual differences in basic physiological and neurological responses [41–43]. The first represents the neurological threshold for detecting sensory stimuli. The second represents behavioural responses to those sensations (e.g. seeking or avoiding). Here we aimed to test whether the dimension of neurological threshold across all senses is associated with PPPD symptoms. To avoid confusion with objectively measured sensory thresholds, we will refer to this as ‘multi-sensory processing’. First, we simply compared multi-sensory processing across patients and controls. Then we used structural equation modelling (SEM) to investigate the relationship with symptoms of PPPD in the general population.

If this cross-sensory association exists, it is then important to know whether it is mediated by anxiety, because of previous associations found between anxiety and the threshold dimension of Dunn’s model [44–46]. Similarly, it becomes important to know if any association only exists for migraine sufferers, because of migraine’s association with both PPPD symptoms and sensory overload or discomfort [1, 2, 14, 27, 28]. We extended the SEM models to explore these possibilities.

## Method

### Participants

#### Patients with PPPD

Twenty-nine patients were recruited from the vestibular clinic at University Hospital Wales (UHW). All patients had received a diagnosis of PPPD from a Clinical Scientist in Audiology or a Consultant Audiovestibular Physician, following common tests to examine vestibular functioning, including Halmagyi bedside head thrust testing, Video Head Impulse testing (VHIT using Synapsys system), Videonystagmography (typically saccades, pursuit, gaze using GN system) and (sometimes) caloric testing if deemed necessary. The average age of participants was 44 (sd = 14.3, range 11–67), 60% were female.

### General population cohort

Two methods were used to recruit participants from the general population: (1) A community health participant list in Wales, (2) Prolific academic, a website where members of the general public can sign up to take part in studies in return for payment.

Participants in a community health list were emailed an advert and link to take part in a survey. The survey was advertised as being about ‘Health and the Senses’ and contained the following text: ‘The School of Psychology at Cardiff University are investigating health and the senses through an online survey. Dizziness is common in the general population and can have serious consequences for daily functioning and health. The research team are interested in a particular type of dizziness that is triggered by being in certain environments. These tend to be environments where there is a lot of clutter, for example, a supermarket or a crowded street. They are interested in how common this dizziness is in the general population and how it might relate to other conditions (e.g. migraines). In the future, they hope this research will help them to develop more effective rehabilitation tools for dizziness. The online survey will include questions and pictures about sensory sensitivity, dizziness and migraines, and is open to everyone. They would like to hear from a range of people, whether or not you suffer from dizziness and migraines.’ We emphasised the inclusivity of the survey so that individuals with an interest in dizziness and migraines would not selectively participate.

From the 18,683 email addresses sent the invite, we received ~ 2500 responses, 972 of which had complete data for each of our measures (necessary to build the full SEM model, and we use the same sample of participants at each stage for direct comparability). The average age of participants was 57 (sd = 13.8, range 19–86), 72% were female. The median level of education attainment was 3, IQR 2–4 (where 0 = no education, 1 = GCSE/O Level, 2 = A-level/BTEC, 3 = Undergraduate, 4 = postgraduate).

To augment this sample, we used Prolific Academic to recruit 211 participants online in return for £5 compensation. Of these, we received 135 valid responses with data on each measure. The average age of these participants was 27 (sd = 7.1, range 18–55), 27% were female. The median level of education attainment was 3, IQR = 2–3.

Combining the two cohorts provided a sample size of 1107, with a mean age of 56 (sd = 16.47, range 18–86), 67% female, and median education attainment of 3 (IQR 2–4). We asked participants to report if they had a current diagnosis of any common vestibular related conditions (*N* = 111), the summary of which is shown in Table 1. For the comparison with patients, we removed from the ‘non-clinical’ control cohort participants who had reported vestibular conditions, resulting in a sample size of 996. For the SEM analysis (where we are interested in symptom variance in the population rather than having a ‘non-clinical’ control group) we included all the participants from the general population cohort (*N* = 1107), but did not include the PPPD patients we had recruited from the clinic.

**Table 1:** Self-reported vestibular related conditions in the general population cohort (i.e. separate to the 29 patients recruited through clinic)

	*N*	Percent
PPPD	11	0.99
Vestibular migraines	43	3.88
Labyrinthitis	16	1.45
Ménière's disease	15	1.36
BPPV	33	2.98
Vestibular neuritis	2	0.18
Stroke	4	0.36
Head trauma	1	0.09
Vestibular schwannoma	4	0.36
Probable migraine	147	13.28

Migraine was determined by the outcome of the Migraine Screening Questionnaire (described in Measures section)

All procedures were approved by the Cardiff and the Vale University Health Board and the School of Psychology, Cardiff University, ethics committees, and have therefore been performed in accordance with the ethical standards laid down in the 1964 Declaration of Helsinki and its later amendments.

### Measures

All aspects of the survey were delivered via Qualtrics, an online survey tool.

*Demographic information:* Basic demographic information was collected including age, gender, educational attainment and if they had a current diagnosis of any common vestibular conditions.

*Visual Vertigo Analogue Scale* [VVAS, 5] was used to evaluate symptoms of PPPD. Participants rated on a scale of 0–10 the amount of dizziness they experience in 9 situations that are known triggers for visually-induced dizziness. These include walking down a supermarket aisle, walking across a patterned floor, and going to the cinema. Scores on each item are typically averaged and then multiplied by ten so that the total score an individual could achieve by rating all situations a 10 (maximum dizziness) is 100. However, in our SEM model, we included the nine items as separate indicators for a ‘PPPD symptoms’ latent factor. The overall internal consistency of these indicators was good (See Fig. 2 for full item loadings and supplementary materials Sections A-C for construct reliability and validity checks).

**Fig. 1:**
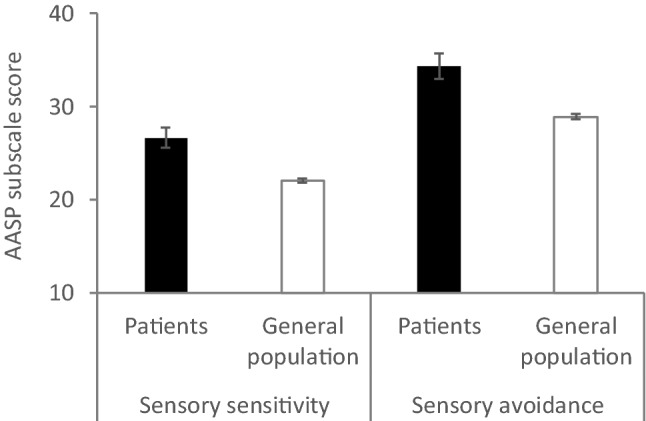
Comparison of the sensory sensitivity and sensory avoidance subscales of the AASP in patients with PPPD (*N* = 29) and members of the general population (*N* = 996).

**Fig. 2:**
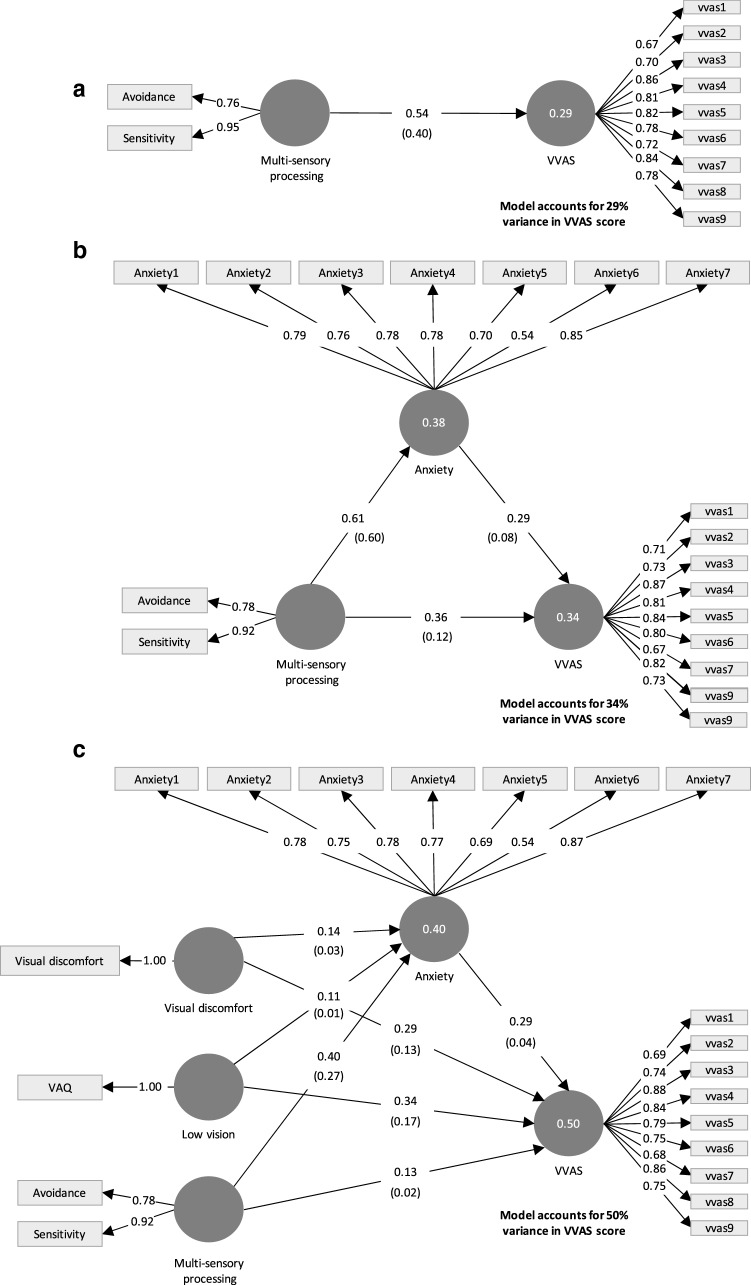
Results of structural equation modelling of the hypothesised mediating role of anxiety on the relationship between multi-sensory processing, visual discomfort, low vision and PPPD symptoms (VVAS scores) in a general population cohort (*N* = 1107). Circles represent the latent factors, and rectangles show indicator variables. Values along the outer model paths show β coefficients, values within brackets show *f*^2^ effect sizes, values within the latent factor circles are *R*^2^. Indicator loadings are shown on arrows connecting latent factors to indicator variables. (Panel **a**) Significant initial relationship between multi-sensory processing and PPPD symptoms. (Panel **b**) Relationship between multi-sensory processing and PPPD symptoms is partially mediated by anxiety. (Panel **c**) Full model that also includes visual discomfort and low vision factors, with mediating paths through anxiety to VVAS (PPPD symptoms)

*Adolescent/Adult Sensory Profile [AASP, 41]:* The adolescent/adult sensory profile (AASP) is a questionnaire that measures individual differences in four sensory subtypes related to Dunn’s model [41]. We were primarily interested in the subscales that assess ‘low sensory threshold’ [47], which are called ‘sensory sensitivity’ and ‘sensory avoidance’ (i.e. behaviours attempting to counteract strong sensations, such as avoiding strong tastes). The AASP is a 60 item questionnaire that asks participants to rate on a Likert scale the frequency that they perform certain behaviours (almost never, seldom, occasionally, frequently and almost always). The questions are split into six sensory domains: taste/smell processing, movement processing, visual processing, touch processing, activity level and auditory processing. Items from the movement processing and visual processing domains were removed as these overlap with known PPPD symptoms. The sensory sensitivity and avoidance subscales showed acceptable internal consistency in our sample (sensory sensitivity Cronbach’s *a* = 0.73; sensory avoidance *a*  = 0.77). Due to the high collinearity between these subscales (*R* = 0.72), the scores were included as indicators to one ‘multi-sensory processing’ latent factor in our SEM model, which again showed good construct reliability and validity (see Fig. 2 for loadings and supplementary materials for inner model checks; note we use the label ‘multi-sensory processing’ to avoid confusion with objectively measured sensory thresholds).

*Hospital Anxiety and Depression Scale [HADS, 48]:* The HADS is a 14 item scale containing 7 questions that contribute to an anxiety subscale and seven questions related to a depression subscale. Due to the previous focus on anxiety and dizziness, we only used the anxiety subscale. Example anxiety questions include: ‘I feel tense or wound up’ and ‘I get a sort of frightened feeling as if something awful is about to happen’. Participants are given four response options per question (.e.g. most of the time, a lot of the time, from time to time, not at all) and are asked to select the option that is closest to how they have been feeling in the past week. Questions are both positively and negatively worded. Options are scored from 0–4, where 4 indicates more anxiety. Items can then be summed to provide an overall anxiety subscale score, however, in our SEM model, we included the seven items as separate indicators to an ‘anxiety’ latent factor. The overall internal consistency of these indicators was good (see Fig. 2 for full item loadings and supplementary materials for construct validity checks).

*Migraine Screening Questionnaire [MS-Q, 49]:* The MS-Q is a five-item screening tool that identifies probable migraine. Participants answer yes/no questions about headache episodes they experience, which include ‘Do you usually suffer from nausea when you have a headache?’ and ‘Does light or noise bother you when you have a headache?’ Participants must respond ‘yes’ to four or more of the five questions to obtain a result of probable migraine.

*Visual Activities Questionnaire [VAQ, 50, 51]:* The VAQ is a measure of low vision difficulties that can affect every-day life across a number of domains, such as acuity, visual search, peripheral vision and colour vision. The original VAQ contains 33 items and 8 subscales [51], however, due to time constraints on participants, we used a reduced 13 item, unidimensional version [50]. Each question asks how often the problem occurs on a 5-category rating scale from 1 (never) to 5 (always). Items are averaged to produce a total score, where higher values indicate more low vision difficulties. The total VAQ score showed good internal consistency in our sample (*a* = 0.86).

*Visual discomfort:* Participants were asked to rate on a scale of 0–10 the amount of discomfort they experienced when viewing a selection of 20 images. These images were taken from Penacchio and Wilkins [52] and we used them previously in our paper on visual discomfort and symptoms of PPPD [28]. Half of these images were ‘high discomfort’ in terms of both previous participant ratings and spectral content analysis. The remaining half were categorised as low discomfort. The images spanned three categories: photographs of buildings, abstract art, and geometric shapes. The images were embedded in the Qualtrics questionnaire and were viewed on participants’ personal devices, so they were rendered at slightly different sizes and resolutions across participants. However, we asked participants at the beginning of the questionnaire to use the device with the biggest screen (e.g. tablet preferable over a phone). Most participants used a computer monitor or a tablet to view the images (computer = 62%, tablet = 21%, phone = 17%). The average resolution (width × height) for the devices used by participants was 1,059,422 pixels. On a standard 22in monitor with a viewing distance of 60 cm, the images subtended 25° × 15° of visual angle. We subtracted average ratings of discomfort on high discomfort images from low discomfort images, to yield a ‘visual discomfort score’. This score represented a participant’s particular aversion images that deviate from the statistical properties of natural scenes.

*Situational characteristics questionnaire [SCQ, 53]:* The SCQ is a 20 item questionnaire that asks participants to rate discomfort in different situations that include intense visual salience or visual-vestibular conflict. It was originally developed to measure space and motion discomfort, but this is now considered to be closely associated with the new diagnosis of PPPD [1]. Situations are rated between 0 and 3 and scores are normalised by subtracting responses to paired situations that are not commonly associated with visually-induced dizziness. The final score is obtained by dividing the summed ratings across all items by the total number of items and multiplying by 10, therefore the maximum score that can be given is 30. Item 15 from the Prolific academic responses was removed due to a question transcription error. We have previously shown that the VVAS has higher internal consistency than the SCQ in a general population sample [27], and so we used the SCQ as a secondary outcome measure of PPPD symptoms and report the results in the Supplementary materials (Section D). Due to a large number of items in the SCQ (*N* = 20), items were combined following standard procedures to produce one SCQ score, and this was included as the outcome factor in our SEM model.

### Structural equation modelling (SEM) procedure

Data were analysed using Smart-PLS, a software that supports partial least squares, or component-based, SEM [54, 55]. In the PLS routine, indicator variables (e.g. questionnaire items or scores) are first standardised to a mean of 0 and a standard deviation of 1. Next, the standardised indicator variables are combined and equally weighted to produce latent variables scores (using a ‘reflective’ model, which assumes indicators, such as questionnaire components, reflect rather than cause the underlying construct, such as anxiety). Initial weights are then applied to the hypothesised paths between the latent variables in such a way that maximises the *R*-squared of each latent variable. After this initial estimation, the PLS algorithm iteratively adjusts the weighting of the indicators and the latent variable path connections to maximise the explained variance across the model. These iterations stop when there is no significant change in the weights of the indicator variables.

Before interpreting the results from the SEM, we carried out a number of checks to ensure the validity of the outer (indicator to latent variables) and inner models (latent variable paths) [54, 55]. We first checked the construct validity of the indicator relationships to each latent variable by examining indicator reliability (average loading > 0.7), internal consistency (Cronbach *a* > 0.7), convergent validity (average variance explained, AVE > 0.5) and discriminant validity (Heterotrait-Monotrait Ratio, HTMT < 0.85). If these were within the limits of acceptability, we then checked that none of the latent variables was collinear (VIP < 5). We then used bias-corrected accelerated bootstrapping (5000 iterations) to test the significance of each path coefficient and report the *F* square as a measure of effect size (where *f*^2^ > 0.3 = large effect, *f*^2^ > 0.15 moderate effect, *f*^2^ < 0.15 = small effect). Due to the moderately large sample size, even small coefficients tended to be significant, so it was important to examine the *f*^2^ values. Finally, we used a blindfolding technique to ensure that estimated model was robust by iteratively removing a subset of the cases and calculating the predictive accuracy of the reduced data model to estimate the omitted data points (*Q*^2^ > 0).

The main predicted variable in all models was PPPD symptoms. In the main analysis we used the VVAS as our measure of PPPD. We repeated all path models using the SCQ as our measure of PPPD and report these results in the supplementary materials.

## Results

### Patient cohort comparison

Our first analysis of interest was whether patients with a diagnosis of PPPD would report differences in multi-sensory processing when compared to members of the general population. We were interested in the two subscales for low neurological threshold—sensory sensitivity and avoidance. We removed questions related to vestibular and visual processing from the AASP as we wanted to examine differences in other sensory modalities. For this analysis, we excluded any members of the general population sample who reported vestibular conditions (*N* = 111), to create a non-clinical comparison group, resulting in an N of 996. Using an ANVOCA and controlling for age and gender, we found that patients with PPPD reported increased indicators of sensory sensitivity and sensory avoidance compared to members of the general population (Fig. 1; sensory sensitivity, *F* (1982) = 10.67, *p* < 0.001; sensory avoidance, *F* (1982) = 11.16, *p* < 0.001).

### Association between multi-sensory processing and PPPD symptoms in the general population

In the next stages of the analysis we used structural equation modelling (SEM) to explore the relationship between multisensory processing and symptoms of PPPD in more depth, using our large general population sample. In the first stage of the analysis, we sought to confirm that multi-sensory processing was also associated with PPPD symptoms in the general population cohort. Sensory avoidance and sensory sensitivity, the two low neurological threshold subscales of the AASP, were combined to form one latent factor representing multi-sensory processing. As described in the methods, items related to visual processing and movement processing were removed. The 9 VVAS items were included as indicators to the latent factor of PPPD symptoms. Figure 2a shows this basic model; individual differences in multi-sensory processing were able to explain 29% of the variance in PPPD symptoms (*β* = 0.54, *t* = 19.33, *p* < 0.001, *f*^2^ = 0.40). The outer and inner models both passed the validity checks described in the methods (full results reported in supplementary materials Section A).

### Mediation effect of anxiety on relationship between multi-sensory processing and PPPD symptoms in the general population

In the next stage of our analysis, we tested the prediction that anxiety would mediate the relationship between multisensory processing and PPPD symptoms. Figure 2b shows that this prediction was supported by the data (outer and inner models validity checks were all acceptable and are reported in supplementary materials Section B). Multi-sensory processing was significantly associated with anxiety *β* = 0.61, *t* = 24.49, *p* < 0.001, *f*^2^ = 0.60) with large effect size, and anxiety was significantly associated with PPPD (*β* = 0.29, *t* = 6.88, *t* < 0.001, *f*^2^ = 0.08), but with a small effect. Bootstrapping revealed that the indirect effect of multisensory processing on PPPD symptoms through anxiety was statistically significant *β* = 0.18, *t* = 6.68, *p* < 0.001). However, the direct effect between multi-sensory process and PPPD symptoms remained significant, although reduced in size (*β* = 0.36, *t* = 8.79, *p* < 0.001, *f*^2^ = 0.12), consistent with a partially mediated effect.

These results suggest that multi-sensory processing retains an association with PPPD symptoms that is independent of anxiety. The model with anxiety was able to explain 34% of the variance in PPPD symptoms.

### Full model including visual discomfort and low vision factors

Next, we included the visual discomfort and low vision factors which have previously been associated with PPPD symptoms and general dizziness, respectively [28, 34]. We were interested in whether their relationship with PPPD, like multi-sensory processing, was partially mediated by anxiety, and whether these visual factors would still be predictive of PPPD symptoms once anxiety was taken into account. Figure 2c shows this full model (outer and inner models validity checks were all acceptable and are reported in supplementary materials Section C).

When visual discomfort and low vision were included in the model, the factors were able to explain 50% of the variance in PPPD symptoms, an increase of 16% over the sensory processing and anxiety only model. This is a moderate effect [55] and is reasonably surprising given that no vestibular or proprioceptive measures were included in the model, which would also be likely to explain some variance in PPPD symptoms.

The direct effect between visual discomfort and PPPD symptoms was significant, although small (*β* = 0.29, *t* = 7.51, *p* < 0.001, *f*^2^ = 0.13). There was a medium, significant direct effect between low vision and PPPD symptoms *β* = 0.34, *t* = 7.83, *p* < 0.001, *f*^2^ = 0.17). The indirect effect of visual discomfort on PPPD symptoms through anxiety was also significant (*β* = 0.03, *t* = 3.17, *p* < 0.01), however, the effect size of the relationship between visual discomfort and anxiety was, while significant, very small (*β* = 0.14, *t* = 4.30, *p* < 0.001, *f*^2^ = 0.03). Likewise, the indirect effect of low vision on PPPD symptoms through anxiety was also significant (*β* = 0.02, *t* = 2.54, *p* < 0.05), but again the path coefficient between low vision and anxiety, although significant, was very small (*β* = 0.11, *t* = 3.10, *p* < 0.01, *f*^2^ = 0.01). These results suggest that anxiety has a small, partial mediating effect on the relationships between visual discomfort, low vision and PPPD symptoms.

To summarise, both visual discomfort and low vision were associated with increased PPPD symptoms. There was a small mediating effect of anxiety but both direct associations remained when taking anxiety into account.

### Comparison of model paths between participants with and without migraine

In the final stage of the analysis, we investigated whether the relationships we observed between the latent factors in our model were the same for participants with and without migraine (i.e. if migraine moderated any of the path coefficients). We wanted to ensure that our results were not explicable based on the inclusion of the 13% of participants with migraine. A multi-group analysis was conducted whereby the model was analysed separately for participants with and without migraine, and bootstrapping was used to compare the path coefficients across the two groups.

The relationship between multi-sensory processing and PPPD was not statistically different between the groups (migraine *β* = 0.15, no migraine *β* = 0.10, *t* = 0.63, *n.s.*). Neither was the relationship between anxiety and PPPD (migraine *β* = 0.08, no migraine *β* = 0.18, *t* = 1.11, n.s.). However, the relationship between multisensory processing and anxiety was significantly stronger for those with migraine than those without (migraine *β* = 0.63, no migraine *β* = 0.35, *t* = 3.06, *p* < 0.01). The mediation effect of anxiety on the relationship between multisensory processing and PPPD symptoms was not moderated by migraine (migraine indirect effect *β* = 0.05, no migraine indirect effect *β* = 0.06, *t* = 0.38, *n.s.*).

The relationship between visual discomfort and PPPD symptoms was not significantly moderated by migraine (migraine symptoms was not significantly *β*  = 0.27, no migraine *β* = 0.29, *t* = 0.24, *n.s.*) and neither was the relationship between low vision and PPPD symptoms (migraine *β* = 0.44, no migraine *β* = 0.30, *t* = 1.02, *n.s*.). However, the relationship between visual discomfort and anxiety was stronger in participants without migraine (migraine *β* = − 0.06, no migraine *β* = 0.17, *t* = 2.49, *p* < 0.05). The relationship between low vision and anxiety was not statistically different between the groups (migraine *β* = 0.00, migraine *β* = 0.16, *t* = 1.65, *n.s*.). The partial mediation effect of anxiety on the relationship between visual discomfort and PPPD was not moderated by migraine (migraine indirect effect = 0.00, no migraine indirect effect = 0.03, *t* = 1.57, *n.s*.) and neither was the relationship between low vision and PPPD (migraine indirect effect *β* = 0.00, no migraine indirect effect, *β* = 0.03, *t* = 1.31, *n.s*.).

In summary, most of the path coefficients and mediation effects in our SEM model were unchanged between participants with and without migraine. However, we found some evidence that the relationship between anxiety and multi-sensory processing was stronger for participants with migraine than those without, whereas the relationship between anxiety and visual discomfort was weaker in those with migraine than those without.

## Discussion

Patients presenting with PPPD in neurology and neuro-otology clinics often anecdotally report feelings of sensory overload. We hypothesised that PPPD might be related to global differences in multi-sensory processing that go beyond vision and vestibular processing. To summarise our results, we first found that patients with PPPD reported greater indicators of low neurological threshold across multiple senses than do members of the general population, even though questions relating to vision and vestibular functioning were removed from the multi-sensory questionnaire.

We then found the same relationship within the general population cohort, in the form of a strong correlation between indicators of multi-sensory threshold and PPPD symptoms. Thus, individuals with increased PPPD symptoms also report increased sensitivity and avoidance behaviours across a range of sensory modalities, including touch, taste, smell and audition.

In the same general population cohort, we next found that this relationship was partially mediated by anxiety. Correlations between PPPD symptoms and visual discomfort and low vision were also partially mediated by anxiety. In our final model, multi-sensory processing, anxiety, visual discomfort and low vision, were together able to explain 50% of the variance in PPPD symptoms measured by VVAS scores; and 40% when measured by SCQ scores (see Supplementary materials section D). Given that no vestibular or proprioceptive measures were included in the model, or even questions about visual motion, this suggests that sensory and psychogenic factors are more influential than previously accounted for in theories of PPPD.

Lastly, all the main findings occurred irrespective of co-morbid migraine, indicating that migraine is not the main reason for these associations, and does not even moderate them.

### Multi-sensory processing and PPPD

The measure of multi-sensory processing (AASP) contains questions across multiple senses. We removed questions that were related to vision and vestibular processing, so our results suggest that people with more symptoms of PPPD have lower sensory thresholds (or at least stronger sensory experiences) across other sensory modalities not usually associated with dizziness. In turn, we infer that touch, taste, smell and auditory sensations may be part of what is driving reported experiences of ‘sensory overload’. It might, therefore, be useful for clinicians to ask patients with PPPD if they find other sensory modalities challenging in their day-to-day lives (and to develop a focussed questionnaire for this). Heightened vulnerability to sensory overload may make individuals more vulnerable to severe or long-lasting PPPD when faced with additional vestibular disruption. In addition, it may be that experiencing challenges in one modality can sometimes exacerbate challenges in other modalities.

The cross-modal aspect may represent a generalised predisposition of some brains to sensory overload, but we do not yet know what mechanisms could account for this. We believe an avenue to investigate this would be to start with the relationship between PPPD symptoms and visual discomfort to highly salient and cluttered images [28]. These images deviate from natural scene statistics and appear to overload the visual system both in imaging studies and computational models [29, 52, 56–59]. Therefore, in the visual modality, we have a theoretical approach to overload that is less well developed in other modalities. It will be interesting to explore whether acoustic, taste and smell overload is greater for stimuli that deviate from natural environments.

### The role of anxiety and other psychogenic factors

Consistent with the known association between anxiety and PPPD, and between anxiety and sensory sensitivity/avoidance in other conditions [44–46], we found that anxiety partially mediated the correlation between PPPD symptoms and multi-sensory processing. The direction of the causal relationship, however, remains unknown. Both causal directions are theoretically plausible. A lower neurological threshold to sensory information could lead to anxiety because the sensory world is overwhelming [e.g. 44]. Alternatively, increased anxiety could lead to over-reactivity to incoming sensory information [60], perhaps via recurrent amplification or boosting of sensory responses [61–63].

Clinically, anxiety is easier to treat using pharmacological and psychological therapies than over-sensitivity to sensory information. It would, therefore, seem most viable to target the anxiety symptoms and hope for a reduction in the sensory symptoms. Pharmacological agents that are commonly used in the treatment of anxiety and depression, such as selective serotonin reuptake inhibitors (SSRIs), have also been found to be effective in treating some types dizziness [64]. Emotion, anxiety and balance systems appear to share some common neural pathways [65, 66] and may meet in the parabrachial nucleus in the dorsolateral pons [67]. Taken together, the relationship between PPPD and anxiety supports the idea that a large component of the condition is driven by factors beyond the inner ear.

However, we also found a strong relationship between multi-sensory processing and PPPD symptoms that was independent of anxiety. Therefore, treating anxiety symptoms would not be expected to completely resolve the multi-sensory processing atypicality. Future research could explore ways of desensitising individuals to multi-sensory stimulation and investigate whether this leads to any improvement in PPPD symptoms.

Many of the features of chronic PPPD bear similarities to the concept of symptom amplification found in functional somatic syndromes [68]. In this framework, individuals with somatic syndromes develop intense self-scrutiny of bodily sensations, which they then attribute to their condition. This results in a self-perpetuating seeking of confirmatory evidence for their ailment, even without medical evidence of physiological dysfunction. It is known that patients with PPPD tend to be over-vigilant about vestibular sensations, which may exacerbate symptoms. Symptom amplification could also be associated with differences in multisensory processing, and it would be interesting to examine the connection between these two constructs in future work.

### Low vision, anxiety and PPPD

In our final model, we included visual factors related to low vision and visual discomfort. Visual discomfort has been discussed above in relation to sensory overload. Low visual abilities have previously been associated with general levels of dizziness [34], and here we find that they are linked to PPPD symptoms in a general population and that this is partially mediated by anxiety (though only with small effect sizes). The visual dependence theory of PPPD [7–11] would offer the explanation that low vision could exacerbate PPPD symptoms if individuals are more reliant on vision for postural control and stability. Partial mediation via anxiety could arise because problems with eyesight would make navigating the world more worrying. One limitation is that we used a self-reporting questionnaire to measure low vision ability. Optical tests would have increased the validity.

### Comorbid migraine

It was important to show that the associations we found existed without co-morbid migraine because migraine has a long-known relationship with PPPD [1, 2, 14]. Further, it is still debated whether patients with PPPD and comorbid migraine (where PPPD-like symptoms might arise via migraine mechanisms) are qualitatively different to those with PPPD without migraine. We were therefore interested in whether the models differed between participants with and without migraine. We found that results were largely the same for both groups, so there is no evidence here for a qualitative difference between groups.

One minor result that differed was that relationship between multi-sensory processing and anxiety was significantly stronger in those with migraine than those without. This might arise if sensory overload sometimes triggers migraine attacks and this raises general anxiety. Second, the relationship between visual discomfort and anxiety was weaker in those with migraine. This might occur simply if the variance in visual discomfort is smaller in the migraine group, as might be expected because visual discomfort is generally raised in migraine sufferers.

### Caveats

The study used an email-based advert to recruit participants from a community sample in the general population, and uptake was relatively low (~ 2500 responses from 18,683 potential participants, with 972 providing complete data on all measures). This is not unexpected for several reasons. Many participants sign up to mailing lists but then decide not to participate or miss emails. Even if they saw the advert, they did not receive compensation for taking part and the survey was relatively time-consuming (~ 25 min). There is always the possibility of response bias where participants with an interest in dizziness or sensory sensitivity may have self-selected to take part. However, we have also collected data from a student population (not reported in this manuscript) who took part in return for course credit and did not receive an advert for the study. This cohort actually showed higher self-reported PPPD symptoms than the community cohort [27], whereas if there were strong self-selection in the community cohort we would expect this to be the other way around.

A second limitation is that the study relied on self-reported measures, and therefore we must trust participants’ introspective abilities. It could be that some participants are more likely to provide higher ratings on all questionnaires, while others are more likely to provide low ratings in all scales, which would over-inflate the relationships in the model. However, we replicated all of the findings using the SCQ as a measure of PPPD symptoms, and the SCQ uses difference scores rather than absolute ratings to minimise response bias effects. Furthermore, we did not find a relationship between factors that we did not expect to be related to PPPD symptoms, such as sensory seeking, which acts as a validity check for our methods (see Supplementary Materials Section E). Finally, all of the factors in the model showed good internal consistency and construct validity, which suggests that the questionnaire data quality was acceptable.

We did not have the sample size of patients with a clinical diagnosis of PPPD to further explore the simple result that patients reported higher multi-sensory sensitivity/avoidance than controls. In patients, we could not examine mediation via anxiety or moderation by migraine. It is still possible that sub-clinical symptoms of PPPD are qualitatively different from diagnosed cases of PPPD, which might limit the transferability of the SEM model findings. We did replicate all the previously known relationships between PPPD and factors such as migraine and anxiety in our non-clinical sample, which suggests that at least in this respect our non-clinical cohorts were like patients with PPPD.

### Summary

We report the novel finding that sufferers of PPPD symptoms also report higher indicators of sensory sensitivity and avoidance across other senses, including touch, hearing, taste and smell. This may represent a lower neurological threshold for sensory stimuli. This relationship was only partially mediated by anxiety. We conclude that experiences of sensory overload in PPPD extend beyond vision and may be associated with a pre-existing cross-model physiological difference in perceptual processing. The mechanism for this interpretation is unknown but may be revealed by studying visual overload in the first instance.

Further, we found that adding factors of self-reported low vision and visual discomfort allowed the model to explain 50% of PPPD symptom variance. Current theories of PPPD do not fully account for this, given that our model did not contain any vestibular or proprioception measures, or questions about visual motion. Our findings support the idea that PPPD is a complex, neurological condition that includes both psychogenic and perceptual factors.

## Electronic supplementary material

Below is the link to the electronic supplementary material.

Supplementary file1 (DOCX 62 kb)
