# Supplementary material for: Visually-induced dizziness is associated with sensitivity and avoidance across all senses
Source: J Neurol. 2020 Apr 18;267(8):2260–71. doi: 10.1007/s00415-020-09817-0 (PMC7359147; doi:10.1007/s00415-020-09817-0)
Supplement: Supplementary file 1 — Supplementary file1 (DOCX 62 kb) [file 415_2020_9817_MOESM1_ESM.docx]

Visually-induced dizziness is associated with sensitivity and avoidance across all senses

**Inner and outer model validity checks**

Following Chin (1998) and Garson (2016), we used the following tests and criterions to check the validity of the inner and outer models:

|  | **Test** | **Criterion** |
| --- | --- | --- |
| Internal consistency | Cronbach Alpha | > 0.7 |
| Convergent validity | Average variance explained (AVE) | > 0.5 |
| Discriminant validity | Heterotrait-Monotrait Ratio (HTMT) | < 0.85 |
| Latent variable collinearity | VIP | < 5 |
| Model predictive power | Blindfolding, Q² | > 0 |

All of the models we report in the main text were within these limits of acceptability, however, exact values for each test are reported below.

**Section A. Stage 1 multi-sensory alone predicts VVAS**

|  | **Cronbach's Alpha** | **AVE** |
| --- | --- | --- |
| Multi-sensory processing | 0.837 | 0.739 |
| VVAS | 0.932 | 0.605 |

*Supplementary Table A1.* Internal consistency and convergent validity of latent factors, Multi-sensory processing and VVAS.

|  | **HTMT** | **VIP** |
| --- | --- | --- |
| Multi-sensory processing & VVAS | 0.537 | 1 |

*Supplementary Table A2.* Discriminant validity and collinearity between Multi-sensory processing and VVAS.

|  | **Q²** |
| --- | --- |
| VVAS | 0.14 |

*Supplementary Table A3.* Results from blindfolding procedure showing good predictive power of the SEM model, even when a select number of cases are removed.

|  | **Avoidance** | **Sensitivity** | **vvas1** | **vvas2** | **vvas3** | **vvas4** | **vvas5** | **vvas6** | **vvas7** | **vvas8** | **vvas9** |
| --- | --- | --- | --- | --- | --- | --- | --- | --- | --- | --- | --- |
| Avoidance | 1.00 | 0.72 | 0.28 | 0.27 | 0.34 | 0.32 | 0.35 | 0.31 | 0.28 | 0.36 | 0.31 |
| Sensitivity | 0.72 | 1.00 | 0.33 | 0.37 | 0.44 | 0.42 | 0.41 | 0.40 | 0.38 | 0.42 | 0.40 |
| vvas1 | 0.28 | 0.33 | 1.00 | 0.45 | 0.66 | 0.60 | 0.85 | 0.60 | 0.49 | 0.59 | 0.56 |
| vvas2 | 0.27 | 0.37 | 0.45 | 1.00 | 0.56 | 0.57 | 0.49 | 0.53 | 0.57 | 0.53 | 0.56 |
| vvas3 | 0.34 | 0.44 | 0.66 | 0.56 | 1.00 | 0.63 | 0.71 | 0.59 | 0.54 | 0.68 | 0.62 |
| vvas4 | 0.32 | 0.42 | 0.60 | 0.57 | 0.63 | 1.00 | 0.74 | 0.60 | 0.59 | 0.64 | 0.64 |
| vvas5 | 0.35 | 0.41 | 0.85 | 0.49 | 0.71 | 0.74 | 1.00 | 0.64 | 0.55 | 0.66 | 0.61 |
| vvas6 | 0.31 | 0.40 | 0.60 | 0.53 | 0.59 | 0.60 | 0.64 | 1.00 | 0.53 | 0.65 | 0.54 |
| vvas7 | 0.28 | 0.38 | 0.49 | 0.57 | 0.54 | 0.59 | 0.55 | 0.53 | 1.00 | 0.58 | 0.74 |
| vvas8 | 0.36 | 0.42 | 0.59 | 0.53 | 0.68 | 0.64 | 0.66 | 0.65 | 0.58 | 1.00 | 0.65 |
| vvas9 | 0.31 | 0.40 | 0.56 | 0.56 | 0.62 | 0.64 | 0.61 | 0.54 | 0.74 | 0.65 | 1.00 |

*Supplementary Table A4.* Correlations between indicator variables included in the model.

**Section B. Stage 2 The relationship between Multi-sensory processing and VVAS is partially mediated by anxiety**

|  | **Cronbach's Alpha** | **AVE** |
| --- | --- | --- |
| Anxiety | 0.893 | 0.555 |
| Multi-sensory processing | 0.837 | 0.73 |
| VVAS | 0.932 | 0.605 |

*Supplementary Table B1.* Internal consistency and convergent validity of latent factors, Multi-sensory processing, anxiety and VVAS.

|  | **HTMT** | **VIP** |
| --- | --- | --- |
| Anxiety & Multi-sensory processing | 0.621 | 1 |
| Anxiety and VVAS | 0.509 | 1.604 |
| Multi-sensory processing & VVAS | 0.537 | 1.604 |

*Supplementary Table B2.* Discriminant validity and collinearity between Multi-sensory processing, anxiety and VVAS.

|  | **Q²** |
| --- | --- |
| Anxiety | 0.167 |
| VVAS | 0.177 |

*Supplementary Table B3.* Results from blindfolding procedure showing good predictive power of the SEM model, even when a select number of cases are removed.

|  | **Avoidance** | **Sensitivity** | **anxiety1** | **anxiety2** | **anxiety3** | **anxiety4** | **anxiety5** | **anxiety6** | **anxiety7** | **vvas1** | **vvas2** | **vvas3** | **vvas4** | **vvas5** | **vvas6** | **vvas7** | **vvas8** | **vvas9** |
| --- | --- | --- | --- | --- | --- | --- | --- | --- | --- | --- | --- | --- | --- | --- | --- | --- | --- | --- |
| Avoidance | 1.00 | 0.72 | 0.37 | 0.37 | 0.40 | 0.39 | 0.34 | 0.29 | 0.39 | 0.28 | 0.27 | 0.34 | 0.32 | 0.35 | 0.31 | 0.28 | 0.36 | 0.31 |
| Sensitivity | 0.72 | 1.00 | 0.43 | 0.42 | 0.43 | 0.45 | 0.39 | 0.31 | 0.46 | 0.33 | 0.37 | 0.44 | 0.42 | 0.41 | 0.40 | 0.38 | 0.42 | 0.40 |
| anxiety1 | 0.37 | 0.43 | 1.00 | 0.60 | 0.61 | 0.63 | 0.54 | 0.35 | 0.62 | 0.32 | 0.34 | 0.37 | 0.33 | 0.35 | 0.33 | 0.26 | 0.33 | 0.28 |
| anxiety2 | 0.37 | 0.42 | 0.60 | 1.00 | 0.67 | 0.58 | 0.64 | 0.38 | 0.70 | 0.28 | 0.29 | 0.33 | 0.31 | 0.33 | 0.34 | 0.23 | 0.32 | 0.25 |
| anxiety3 | 0.40 | 0.43 | 0.61 | 0.67 | 1.00 | 0.59 | 0.57 | 0.37 | 0.66 | 0.28 | 0.30 | 0.34 | 0.32 | 0.35 | 0.30 | 0.24 | 0.31 | 0.26 |
| anxiety4 | 0.39 | 0.45 | 0.63 | 0.58 | 0.59 | 1.00 | 0.50 | 0.42 | 0.58 | 0.30 | 0.30 | 0.33 | 0.30 | 0.32 | 0.31 | 0.24 | 0.30 | 0.24 |
| anxiety5 | 0.34 | 0.39 | 0.54 | 0.64 | 0.57 | 0.50 | 1.00 | 0.30 | 0.68 | 0.25 | 0.26 | 0.31 | 0.31 | 0.30 | 0.30 | 0.21 | 0.26 | 0.24 |
| anxiety6 | 0.29 | 0.31 | 0.35 | 0.38 | 0.37 | 0.42 | 0.30 | 1.00 | 0.42 | 0.18 | 0.20 | 0.22 | 0.18 | 0.24 | 0.18 | 0.15 | 0.22 | 0.20 |
| anxiety7 | 0.39 | 0.46 | 0.62 | 0.70 | 0.66 | 0.58 | 0.68 | 0.42 | 1.00 | 0.35 | 0.34 | 0.41 | 0.40 | 0.40 | 0.38 | 0.28 | 0.36 | 0.31 |
| vvas1 | 0.28 | 0.33 | 0.32 | 0.28 | 0.28 | 0.30 | 0.25 | 0.18 | 0.35 | 1.00 | 0.45 | 0.66 | 0.60 | 0.85 | 0.60 | 0.49 | 0.59 | 0.56 |
| vvas2 | 0.27 | 0.37 | 0.34 | 0.29 | 0.30 | 0.30 | 0.26 | 0.20 | 0.34 | 0.45 | 1.00 | 0.56 | 0.57 | 0.49 | 0.53 | 0.57 | 0.53 | 0.56 |
| vvas3 | 0.34 | 0.44 | 0.37 | 0.33 | 0.34 | 0.33 | 0.31 | 0.22 | 0.41 | 0.66 | 0.56 | 1.00 | 0.63 | 0.71 | 0.59 | 0.54 | 0.68 | 0.62 |
| vvas4 | 0.32 | 0.42 | 0.33 | 0.31 | 0.32 | 0.30 | 0.31 | 0.18 | 0.40 | 0.60 | 0.57 | 0.63 | 1.00 | 0.74 | 0.60 | 0.59 | 0.64 | 0.64 |
| vvas5 | 0.35 | 0.41 | 0.35 | 0.33 | 0.35 | 0.32 | 0.30 | 0.24 | 0.40 | 0.85 | 0.49 | 0.71 | 0.74 | 1.00 | 0.64 | 0.55 | 0.66 | 0.61 |
| vvas6 | 0.31 | 0.40 | 0.33 | 0.34 | 0.30 | 0.31 | 0.30 | 0.18 | 0.38 | 0.60 | 0.53 | 0.59 | 0.60 | 0.64 | 1.00 | 0.53 | 0.65 | 0.54 |
| vvas7 | 0.28 | 0.38 | 0.26 | 0.23 | 0.24 | 0.24 | 0.21 | 0.15 | 0.28 | 0.49 | 0.57 | 0.54 | 0.59 | 0.55 | 0.53 | 1.00 | 0.58 | 0.74 |
| vvas8 | 0.36 | 0.42 | 0.33 | 0.32 | 0.31 | 0.30 | 0.26 | 0.22 | 0.36 | 0.59 | 0.53 | 0.68 | 0.64 | 0.66 | 0.65 | 0.58 | 1.00 | 0.65 |
| vvas9 | 0.31 | 0.40 | 0.28 | 0.25 | 0.26 | 0.24 | 0.24 | 0.20 | 0.31 | 0.56 | 0.56 | 0.62 | 0.64 | 0.61 | 0.54 | 0.74 | 0.65 | 1.00 |

*Supplementary Table B4.* Correlations between indicator variables included in the model.

**Section C. Stage 3 Full model including visual discomfort and low vision factors**

|  | **HTMT** | **VIP** |
| --- | --- | --- |
| Anxiety & Multi-sensory processing | 0.621 | 1.54 |
| Anxiety and VVAS | 0.509 | 1.673 |
| Anxiety and Visual discomfort | 0.399 | 1.275 |
| Anxiety and Low vision | 0.398 | 1.355 |
| Multi-sensory processing & VVAS | 0.537 | 1.955 |
| Multi-sensory processing & Visual discomfort | 0.456 |  |
| Multi-sensory processing & Low vision | 0.503 |  |
| Visual discomfort and VVAS | 0.518 | 1.306 |
| Low vision & VVAS | 0.562 | 1.374 |
| Visual discomfort & low vision | 0.313 |  |

*Supplementary Table C1.* Discriminant validity and collinearity between multi-sensory processing, anxiety, visual discomfort, Low vision and VVAS.

|  | **Q²** |
| --- | --- |
| Anxiety | 0.191 |
| VVAS | 0.278 |

*Supplementary Table C2.* Results from blindfolding procedure showing good predictive power of the SEM model, even when a select number of cases are removed.

|  | **Avoidance** | **Sensitivity** | **VAS** | **Visual discomfort** | **anxiety1** | **anxiety2** | **anxiety3** | **anxiety4** | **anxiety5** | **anxiety6** | **anxiety7** | **vvas1** | **vvas2** | **vvas3** | **vvas4** | **vvas5** | **vvas6** | **vvas7** | **vvas8** | **vvas9** |
| --- | --- | --- | --- | --- | --- | --- | --- | --- | --- | --- | --- | --- | --- | --- | --- | --- | --- | --- | --- | --- |
| Avoidance | 1.00 | 0.72 | 0.37 | 0.36 | 0.37 | 0.37 | 0.40 | 0.39 | 0.34 | 0.29 | 0.39 | 0.28 | 0.27 | 0.34 | 0.32 | 0.35 | 0.31 | 0.28 | 0.36 | 0.31 |
| Sensitivity | 0.72 | 1.00 | 0.48 | 0.42 | 0.43 | 0.42 | 0.43 | 0.45 | 0.39 | 0.31 | 0.46 | 0.33 | 0.37 | 0.44 | 0.42 | 0.41 | 0.40 | 0.38 | 0.42 | 0.40 |
| VAS | 0.37 | 0.48 | 1.00 | 0.31 | 0.32 | 0.29 | 0.32 | 0.31 | 0.28 | 0.18 | 0.36 | 0.39 | 0.45 | 0.48 | 0.51 | 0.43 | 0.38 | 0.39 | 0.44 | 0.45 |
| Visual discomfort | 0.36 | 0.42 | 0.31 | 1.00 | 0.29 | 0.28 | 0.30 | 0.31 | 0.29 | 0.24 | 0.35 | 0.34 | 0.34 | 0.47 | 0.41 | 0.40 | 0.41 | 0.36 | 0.52 | 0.38 |
| anxiety1 | 0.37 | 0.43 | 0.32 | 0.29 | 1.00 | 0.60 | 0.61 | 0.63 | 0.54 | 0.35 | 0.62 | 0.32 | 0.34 | 0.37 | 0.33 | 0.35 | 0.33 | 0.26 | 0.33 | 0.28 |
| anxiety2 | 0.37 | 0.42 | 0.29 | 0.28 | 0.60 | 1.00 | 0.67 | 0.58 | 0.64 | 0.38 | 0.70 | 0.28 | 0.29 | 0.33 | 0.31 | 0.33 | 0.34 | 0.23 | 0.32 | 0.25 |
| anxiety3 | 0.40 | 0.43 | 0.32 | 0.30 | 0.61 | 0.67 | 1.00 | 0.59 | 0.57 | 0.37 | 0.66 | 0.28 | 0.30 | 0.34 | 0.32 | 0.35 | 0.30 | 0.24 | 0.31 | 0.26 |
| anxiety4 | 0.39 | 0.45 | 0.31 | 0.31 | 0.63 | 0.58 | 0.59 | 1.00 | 0.50 | 0.42 | 0.58 | 0.30 | 0.30 | 0.33 | 0.30 | 0.32 | 0.31 | 0.24 | 0.30 | 0.24 |
| anxiety5 | 0.34 | 0.39 | 0.28 | 0.29 | 0.54 | 0.64 | 0.57 | 0.50 | 1.00 | 0.30 | 0.68 | 0.25 | 0.26 | 0.31 | 0.31 | 0.30 | 0.30 | 0.21 | 0.26 | 0.24 |
| anxiety6 | 0.29 | 0.31 | 0.18 | 0.24 | 0.35 | 0.38 | 0.37 | 0.42 | 0.30 | 1.00 | 0.42 | 0.18 | 0.20 | 0.22 | 0.18 | 0.24 | 0.18 | 0.15 | 0.22 | 0.20 |
| anxiety7 | 0.39 | 0.46 | 0.36 | 0.35 | 0.62 | 0.70 | 0.66 | 0.58 | 0.68 | 0.42 | 1.00 | 0.35 | 0.34 | 0.41 | 0.40 | 0.40 | 0.38 | 0.28 | 0.36 | 0.31 |
| vvas1 | 0.28 | 0.33 | 0.39 | 0.34 | 0.32 | 0.28 | 0.28 | 0.30 | 0.25 | 0.18 | 0.35 | 1.00 | 0.45 | 0.66 | 0.60 | 0.85 | 0.60 | 0.49 | 0.59 | 0.56 |
| vvas2 | 0.27 | 0.37 | 0.45 | 0.34 | 0.34 | 0.29 | 0.30 | 0.30 | 0.26 | 0.20 | 0.34 | 0.45 | 1.00 | 0.56 | 0.57 | 0.49 | 0.53 | 0.57 | 0.53 | 0.56 |
| vvas3 | 0.34 | 0.44 | 0.48 | 0.47 | 0.37 | 0.33 | 0.34 | 0.33 | 0.31 | 0.22 | 0.41 | 0.66 | 0.56 | 1.00 | 0.63 | 0.71 | 0.59 | 0.54 | 0.68 | 0.62 |
| vvas4 | 0.32 | 0.42 | 0.51 | 0.41 | 0.33 | 0.31 | 0.32 | 0.30 | 0.31 | 0.18 | 0.40 | 0.60 | 0.57 | 0.63 | 1.00 | 0.74 | 0.60 | 0.59 | 0.64 | 0.64 |
| vvas5 | 0.35 | 0.41 | 0.43 | 0.40 | 0.35 | 0.33 | 0.35 | 0.32 | 0.30 | 0.24 | 0.40 | 0.85 | 0.49 | 0.71 | 0.74 | 1.00 | 0.64 | 0.55 | 0.66 | 0.61 |
| vvas6 | 0.31 | 0.40 | 0.38 | 0.41 | 0.33 | 0.34 | 0.30 | 0.31 | 0.30 | 0.18 | 0.38 | 0.60 | 0.53 | 0.59 | 0.60 | 0.64 | 1.00 | 0.53 | 0.65 | 0.54 |
| vvas7 | 0.28 | 0.38 | 0.39 | 0.36 | 0.26 | 0.23 | 0.24 | 0.24 | 0.21 | 0.15 | 0.28 | 0.49 | 0.57 | 0.54 | 0.59 | 0.55 | 0.53 | 1.00 | 0.58 | 0.74 |
| vvas8 | 0.36 | 0.42 | 0.44 | 0.52 | 0.33 | 0.32 | 0.31 | 0.30 | 0.26 | 0.22 | 0.36 | 0.59 | 0.53 | 0.68 | 0.64 | 0.66 | 0.65 | 0.58 | 1.00 | 0.65 |
| vvas9 | 0.31 | 0.40 | 0.45 | 0.38 | 0.28 | 0.25 | 0.26 | 0.24 | 0.24 | 0.20 | 0.31 | 0.56 | 0.56 | 0.62 | 0.64 | 0.61 | 0.54 | 0.74 | 0.65 | 1.00 |

*Supplementary Table C3.* Correlations between indicator variables included in the model.

**Section D. Situational characteristics questionnaire SEM analysis**

The SEM models were repeated but with the Situational Characteristics Questionnaire (SCQ, Jacob et al., 1993) as the primary outcome measure rather than the VVAS. The results remained largely consistent and are summarised below. All inner and outer models passed the validity checks.

**Participants**

Due to time constraints on participants, the SCQ was included as an optional questionnaire, which resulted in a smaller sample size of 1081. The average age of participants was 54 (std = 16.5, range 18-86) and 68% were female. The median education level was 3 (IQR 2-4). Self-reported vestibular conditions are shown in Supplementary Table D1.

|  | **N** | **Percent** |
| --- | --- | --- |
| PPPD (visual vertigo) | 10 | 1.00 |
| Vestibular Migraines | 42 | 3.89 |
| Labyrinthitis | 13 | 1.20 |
| Ménière's disease | 14 | 1.30 |
| BPPV | 33 | 3.05 |
| Vestibular Neuritis | 2 | 0.19 |
| Stroke | 4 | 0.37 |
| Head Trauma | 2 | 0.19 |
| Vestibular Schwannoma | 4 | 0.37 |
| Probable migraine | 142 | 13.14 |

*Supplementary Table D1.* Self-reported vestibular related conditions for the SCQ sample participants. Probable migraine was determined by the Migraine Screening Questionairre.

**Stage 1: Multi-sensory alone predicts SCQ**

Multi-sensory processing explained 31% of the variance in SCQ scores ($\beta$ = 0.56, $t$ = 23, $p$ < 0.001, $f^{2}$= 0.45), which replicates the relationship between PPPD symptoms and multi-sensory avoidance and sensitivity found with the VVAS. See Supplementary Figure 1A for a schematic of this model.

**Stage 2: The relationship between Multi-sensory processing and SCQ is partially mediated by anxiety**

Consistent with the VVAS results, the relationship between multi-sensory processing and SCQ was partially mediated by anxiety (see Supplementary Figure 1B). Anxiety was significantly related to SCQ scores ($\beta$ = 0.18, $t$ = 4.61 $p$ < 0.001, $f^{2}$ = 0.03) and bootstrapping revealed that the indirect effect of multi-sensory processing on SCQ scores through anxiety was statistically significant, although small ($\beta$ = 0.11, $t$ = 4.57, $p$ < 0.001). However, the direct effect between multi-sensory processing and SCQ scores remained significant ($\beta$ = 0.45, $t$ = 11.83, $p$ < 0.001, $f^{2}$ = 0.19) , which suggests only partial mediation by anxiety.

The model with anxiety also included explained 33% of the variance in SCQ scores, which is only a 2% increase in the multi-sensory only model. This suggests that, particularly for the SCQ, multi-sensory processing is stronger predictor than anxiety.

**Stage 3: Full model including visual discomfort and low vision factors**

In the final SEM model, we included the low vision and visual discomfort factors in the model and explored whether they were also mediated by anxiety. This model was able to explain 40% of the variance in SCQ scores (see Supplementary Figure 1C), which like the VVAS model is a moderate effect.

The results were mostly consistent with the VVAS model. The direct effect between visual discomfort and SCQ scores was significant ($\beta$ = 0.25, $t$ = 8.02, $p$ < 0.001, $f^{2}$= 0.08), as was the direct effect between low vision and SCQ scores ($\beta$ = 0.14, $t$ = 3.82, $p$ < 0.001, $f^{2}$ = 0.02). The indirect effect of visual discomfort on SCQ scores through anxiety was also significant ($\beta$ = 0.01, t = 2.34, $p$ <0.05), as was the indirect effect of low vision on SCQ scores through anxiety ($\beta$ = 0.01, t = 2.1, $p$ <0.05). However, these indirect effects are quite small, while the direct effects remain fairly large, which suggests that the mediation effect of anxiety was not very substantial. These results suggest that, as with the VVAS, both low vision and visual discomfort are associated with increased SCQ scores, and there is a small, partially mediating effect of anxiety.

**Stage 4: Comparison of model paths between participants with and without migraine**

Next we explored whether the relationships between the factors in our model were the same for participants with and without migraine, by running a multi-groups analysis. In the SCQ sample 13.4% of participants had migraine. Again, results remained consistent with the VVAS model. The direct effect between multi-sensory processing and SCQ scores was not significantly different between the migraine and no migraine groups (migraine $\beta$ = 0.29, no migraine $\beta$ = 0.24, $t$ = 0.64, n.s.). Neither was the relationship between anxiety and SCQ scores (migraine $\beta$ = 0.19, no migraine $\beta$ = 0.12, $t$ = 0.88, n.s.). The mediation effect of anxiety on the relationship between multi-sensory processing and PPPD symptoms was not moderated by migraine (migraine indirect effect $\beta$ = 0.12, no migraine indirect effect $\beta$ = 0.04, $t$ = 1.91, *n.s*.).

The relationship between visual discomfort and SCQ scores was not moderated by migraine (migraine $\beta$ = 0.22, no migraine $\beta$ = 0.27, $t$ = 0.52, *n.s.*) and neither was the relationship between low vision and SCQ scores (migraine $\beta$ = 0.1, no migraine $\beta$ = 0.18, $t$ = 0.77, *n.s*.). The partial mediation effect of anxiety on the relationship between visual discomfort and SCQ scores was not moderated by migraine (migraine indirect effect = -0.01, no migraine indirect effect = 0.02, t = 1.66, *n.s*.) and neither was the relationship between low vision and SCQ scores (migraine indirect effect $\beta$ = 0.00, no migraine indirect effect, $\beta$ = 0.02, $t$ = 0.72, *n.s*.). In summary, consistent with the results from the VVAS model, most of the relationship in the SCQ model were unchanged between participants with and without migraine.

*Supplementary Figure 1.* Results of structural equation modelling of the hypothesised mediating role of anxiety on the relationship between multi-sensory processing, visual discomfort, low vision and SCQ scores. Circles represent the latent factors, and rectangles show indicator variables. Values along the outer model paths show $\beta$ coefficients, values within brackets show $f^{2}$ effect sizes, values within the blue latent factor circles are R^2^. Indicator loadings are shown on arrows connecting latent factors to indicator variables. (Panel A) Significant initial relationship between multi-sensory processing and SCQ scores. (Panel B) Relationship between multi-sensory processing and SCQ scores is partially mediated by anxiety. (Panel C) Full model that also includes visual discomfort and low vision factors, with mediating paths through anxiety to SCQ.

**Section E: Correlations between PPPD measures and AASP subscales**

Spearman correlations between the four subscales of the AASP and the VVAS and SCQ are shown in Supplementary Table E1 and E2.

|  | **Low registration** | **Sensory seeking** | **Sensory sensitivity** | **Sensory avoidance** |
| --- | --- | --- | --- | --- |
| VVAS | .40** | -0.03 | .50** | .41** |
| SCQ | .36** | -0.01 | .50** | .45** |

*Supplementary Table E1*. Correlations between VVAS, SCQ and AASP sensory subscales with questions related to vision and vestibular *removed.* ** correlation is significant at 0.01 level (2-tailed).

|  | **Low registration** | **Sensory seeking** | **Sensory sensitivity** | **Sensory avoidance** |
| --- | --- | --- | --- | --- |
| VVAS | .47** | -0.15** | .61** | .49** |
| SCQ | .42** | -.13** | .62** | .51** |

*Supplementary Table E2*. Correlations between VVAS, SCQ and AASP sensory subscales with questions related to vision and vestibular *included.* ** correlation is significant at 0.01 level (2-tailed).

**References**

Chin, W. W. (1998). The partial least squares approach to structural equation modeling. *Modern methods for business research, 295*(2), 295-336.

Garson, G. D. (2016). Partial least squares: Regression and structural equation models. *Asheboro, NC: Statistical Associates Publishers*.

Jacob, R. G., Woody, S. R., Clark, D. B., Lilienfeld, S. O., Hirsch, B. E., Kucera, G. D., . . . Durrant, J. D. (1993). Discomfort with space and motion: a possible marker of vestibular dysfunction assessed by the situational characteristics questionnaire. *Journal of Psychopathology and Behavioral Assessment, 15*(4), 299-324.
